# EGFR Mutation Detection in Whole Slide Images of Non‐Small Cell Lung Cancers Using a Two‐Stage Deep Transfer Learning Approach

**DOI:** 10.1002/cam4.71249

**Published:** 2025-09-18

**Authors:** Michele Zanoletti, Filippo Ugolini, Laila El Bachiri, Valeria Pasini, Marco Laurino, Francesco De Logu, Eleonora Melissa, Carolina Marchi, Maria Colombino, Daniela Massi, Guido Rindi, Camilla Eva Comin, Giuseppe Palmieri, Antonio Cossu

**Affiliations:** ^1^ Institute of Clinical Physiology National Research Council Pisa Italy; ^2^ Section of Pathological Anatomy Careggi University Hospital Florence Italy; ^3^ Unit of Pathological Anatomy and Histology, Azienda Ospedaliero Universitaria, Department of Biomedical Sciences University of Sassari Sassari Italy; ^4^ Section of Pathological Anatomy, Department of Health Sciences University of Florence Florence Italy; ^5^ Section of Clinical Pharmacology and Oncology, Department of Health Sciences University of Florence Florence Italy; ^6^ Department of Experimental and Clinical Biomedical Sciences “Mario Serio” University of Florence Florence Italy; ^7^ Institute of Genetic and Biomedical Research at the National Research Council Sassari Italy; ^8^ Section of Anatomic Pathology, Department of Life Sciences and Public Health Università Cattolica del Sacro Cuore Rome Italy; ^9^ Anatomic Pathology Unit, Department of Woman and Child Health Sciences and Public Health Fondazione Policlinico Universitario Agostino Gemelli IRCCS Rome Italy; ^10^ Department of Experimental and Clinical Medicine Section of Surgery, Histopathology and Molecular Pathology University of Florence Florence Italy; ^11^ Immuno‐Oncology and Targeted Cancer Biotherapies University of Sassari—Unit of Cancer Genetics, Institute of Genetic and Biomedical Research at the National Research Council Sassari Italy; ^12^ Unit of Pathological Anatomy and Histology, Azienda Ospedaliero Universitaria, Department of Medicine, Surgery and Pharmacy University of Sassari Sassari Italy

**Keywords:** adenocarcinoma, artificial intelligence, CNN, deep learning, digital pathology, EGFR mutation, explainability, non‐small cell lung cancer, WSI

## Abstract

**Background:**

Lung cancer (LC) is the leading cause of cancer death worldwide. Non‐small cell lung cancer is the most frequent and includes adenocarcinoma and squamous cell carcinoma. Currently, LC treatment is based on tumor molecular profiling. LC may display Epidermal Growth Factor Receptor (EGFR) gene mutation. Detecting mutations in the EGFR gene is crucial for the tyrosine kinase inhibitory therapy.

**Methods:**

This study used a computer‐based methodology with two Convolutional Neural Networks (CNNs) based on InceptionResNet‐V2, applied to Whole Slide Images, to distinguish healthy from cancerous tissue and then EGFR mutated tumor tissue samples. We also integrated an Explainable AI technique (Grad‐CAM) to clearly visualize insights into the model's decision‐making process. The analysis was conducted on 259 LC cases collected from three different centers (Florence, Rome, and Sassari).

**Results:**

This methodology achieved an accuracy of 96.17% in distinguishing healthy from cancerous tissue, with specificity of 87.89%, sensitivity of 98.43%, an F1 score of 97.59% and an AUC of 0.99. Additionally, Cohen's Kappa indicated a consistency of 0.7982, and the confusion matrix showed a correct classification rate of 96.2%. For EGFR mutation detection in cancer tissue, slide‐level performance after aggregation reached an accuracy of 76.67% with specificity of 80.77%, sensitivity of 73.53%, an F1 score of 78.12%, a consistency of 0.5583 of Cohen's Kappa and an AUC of 0.77. The confusion matrix showed 76.7% as a correct classification rate.

**Conclusion:**

The two tested CNNs showed potential for assisting LC diagnosis, especially in distinguishing healthy from tumor tissue. While the direct detection of EGFR mutational status remains challenging, the results suggest that relevant predictive signals can still be extracted from routine H&E slides.

## 1 Introduction

Despite progress in medical research, lung cancer (LC) continues to be the leading cause of cancer‐related deaths worldwide. According to the Global Cancer Observatory, it is the second most common cancer after breast cancer, with over 2.2 million new cases annually [1, 2]. Although LC incidence has steadily decreased since 2006 to 2007, by 2.6% per year in men and 1.1% per year in women, it remains high [3]. The 5‐year survival rate for LC in both sexes has been gradually improving, reaching 19.4% [2, 4]. Nevertheless, overall mortality remains high due to the low number of cases detected at an early stage.

Approximately 85% of lung cancers are classified as non‐small cell lung cancer (NSCLC), a broad diagnostic category encompassing several subtypes, with adenocarcinoma (ADC) and squamous cell carcinoma (SCC) being the most common [5]. ADC is the predominant subtype, accounting for 45%–50% of NSCLC cases. The current staging system for NSCLC follows the guidelines of the American Joint Committee on Cancer (AJCC) [6]. Depending on the clinical stage at diagnosis, targeted therapies are available for non‐squamous subtypes and certain patient groups with SCC. These therapies work in synergy with conventional treatments, including chemotherapy, surgery, radiotherapy, and immunotherapy [7]. The advent of targeted therapies has underscored the importance of accurate morphological diagnosis, the immunohistochemical and molecular characterization of NSCLC [8].

The use of targeted therapies in patients with specific oncogenic alterations has resulted in a marked improvement in primary efficacy endpoints and a reduced risk of death compared to those without currently known gene mutations [9, 10]. Meanwhile, recent advances in gene sequencing technologies, particularly the growing application of next‐generation sequencing (NGS), have revealed an increasing number of oncogenic alterations [11].

Targetable molecular alterations have been identified in approximately 60% of lung ADC patients in Western populations and around 80% in those of Asian descent [12]. The most common genetic alterations in lung ADC that can be targeted include activating mutations in Epidermal Growth Factor Receptor (EGFR) and Kirsten Rat Sarcoma Viral Oncogene Homolog (KRAS) [13].

In NSCLC, EGFR mutations are restricted to the first four exons (18–21) of the tyrosine kinase domain, which encodes key segments of the gene [14]. These mutations, which include deletions, insertions, and point mutations, all affect the binding site of the tyrosine kinase domain. The prevalence of EGFR mutations varies according to histotype, ethnicity, and other demographic or pathological factors. Studies have shown that sensitizing EGFR mutations are almost exclusively found in ADCs, with a striking prevalence of 78% in East Asian populations, compared to 10%–16% in other ethnic groups [15, 16]. To date, over 200 distinct EGFR gene alterations have been identified, with the most common being in‐frame deletions in exon 19 and L858R substitutions in exon 21. However, 50%–60% of patients with EGFR‐mutated LC initially treated with first‐ and second‐generation EGFR tyrosine kinase inhibitors (TKIs) may develop the T790M resistance mutation in exon 20, which remains sensitive to the third‐generation TKI, Osimertinib [17]. So, current international guidelines recommend EGFR testing not only in the advanced stages of the disease but also in early stage [18]. At present, EGFR mutation testing in NSCLC is conducted using modern sequencing techniques, predominantly NGS, in most laboratories [19].

Given that diagnostic material typically consists of small biopsy fragments or cytological samples, comprehensive morphological and biological characterization can be challenging. Computer‐aided diagnosis (CAD) systems present a promising alternative, providing detailed information on each tumor while reducing time and costs. Recently, artificial intelligence (AI) has gained attention in digital image processing, particularly in neoplastic pathology [20, 21]. The use of whole slide imaging and faster networks has facilitated the sharing and management of digital slide images for clinical use. Deep learning algorithms, especially convolutional neural networks (CNNs), are the most effective AI methods for processing medical images, including histopathology [22]. Notably, recent developments in deep learning radiomics have demonstrated that combining semantic and radiomic features can enhance both diagnostic interpretability and model performance, particularly in complex tasks like neuroimaging for hepatoma expansion. Such approaches not only aid clinical decision‐making but also support the potential of AI in advancing precision medicine [23]. In parallel, the integration of channel attention mechanisms into CNNs has enhanced deep learning performance in biomedical applications by allowing the models to better focus on the most informative features, thereby improving the prediction of protein and peptide toxicity classification [24].

Thoracic imaging has been the first to benefit from artificial intelligence and deep learning approaches [25, 26]. In this medical field, multiple AI‐based tools have been developed, dedicated to the detection and characterization of nodules in neoplastic settings, and to the quantification, characterization and follow‐up of interstitial lung disease in nonneoplastic settings [27].

Currently, several AI applications have been reported also in the field of LC pathology; these include the distinction between healthy parenchyma and tumor, the detection of lymph node metastases, the count of tumor cells, the determination of the immunohistochemical status of the tumor, the determination of predictive biomarkers including programmed death ligand 1 (PD‐L1) status and genetic mutations, risk stratification, and the prediction of response to treatments. Collectively, these studies highlight the significant potential of AI to enhance diagnostic accuracy and inform treatment strategies in LC pathology [28]. Although the integration of AI algorithms into clinical practice remains a significant challenge to date, the results obtained to date are promising [29, 30, 31, 32].

One of the exciting possibilities of AI in pathology is its potential ability to identify subtle and latent features that the human eye cannot see. For example, several studies have reported that AI can predict biomolecular features from Hematoxylin and Eosin (H&E) stained images in various malignancies. This ability not only improves diagnostic accuracy but also opens new avenues for personalized treatment strategies by identifying critical genetic markers that inform treatment decisions [33, 34].

Researchers are currently exploring alternative methods to determine EGFR status in various solid tumors, utilizing AI systems to analyze CT images and predict EGFR mutation status including the mutated subtype (Del19 and L858R) [35]. Concurrent radiomics and pathology studies on Whole Slide Imaging (WSI) integrate AI‐based approaches into diagnostic pathology [36]. The application of AI to histological image analysis offers significant cost and time savings and the ability to directly identify features, mutations and molecular alterations relevant for therapeutic decisions from a histological slide [37].

In the present study, we propose a two‐stage AI‐based approach that exploits CNNs to distinguish between normal tissue and tumor tissue and to subsequently predict the mutational status of EGFR in NSCLCs, particularly for ADCs. The goal of this study is to introduce a modern diagnostic and predictive tool to associate it with conventional pathology practice. Within a real‐world diagnostic workflow, this tool could serve as an initial rapid prescreening step following H&E slide preparation and scanning, helping to prioritize cases for molecular testing where needed. Such an approach may be especially valuable in settings with limited access to NGS, while also opening the door to more autonomous and morphology‐based diagnostics in the future. Given the substantial heterogeneity in staining and scanning observed across the three contributing centers, we opted to partition the dataset into training, validation, and test sets, independently of institutional origin. This strategy ensured a balanced representation of inter‐center variability throughout model development, thereby enhancing its robustness and mitigating the risk of overfitting to site‐specific characteristics.

## 2 Materials and Methods

### 2.1 Sample Population and Data Set

The study included a retrospective collection of formalin‐fixed paraffin‐embedded (FFPE) lung ADCs (*n* = 259) from the Section of Pathology, Department of Health Sciences, University of Florence, Florence, Italy (*n* = 50); the Center of Pathology, Department of Medicine, Surgery and Drugs, University of Sassari, Sassari, Italy (*n* = 100) and the Center of Pathology of the head and neck, lung, and endocrine system, Department of Women's Health, Child Health and Public Health Sciences, Catholic University of the Sacred Heart, Fondazione Policlinico Universitario A. Gemelli IRCCS Rome, Italy (*n* = 109) covering the period between 2017 and 2021. Both surgical and biopsy samples were utilized.

### 2.2 Whole Slide Imaging

Our expert pathologists (Dr. Comin, Dr. Cossu and Dr. Rindi) performed the histopathological revaluation and confirmed the original diagnosis. Representative histopathological slides of ADCs stained with hematoxylin and eosin (H&E) were anonymized and digitalized at an original magnification of 400 using an Aperio AT2 in a WSI (Leica Biosystems, Wetzlar, Germany). Individual. svs format files were imported into HALO digital imaging analysis software version 4.0 (Indica Labs, Albuquerque, NM). The use of FFPE sections of human samples was approved by the Local Ethics Committee 20753‐bio according to the Helsinki Declaration, as well as by the Committee for the Ethics of the Research and Bioethics of the National Research Council n.12629. All patients were informed of the aims and methodology details about the use of their tissue samples and gave their signed consent.

### 2.3 EGFR Mutation Testing

Formalin‐fixed paraffin‐embedded (FFPE) tumor tissue sections from patients with a histologically proven diagnosis of advanced lung adenocarcinoma and containing at least 70% of malignant cells were prepared for genomic DNA isolation and purification. The extraction of nucleic acids, quantification, and quality assessment of the extracted nucleic acids were performed using standardized procedures. The nucleic acids were then diluted to the specific concentrations required by the NGS panel employed.

Mutation analyses were performed according to the clinical need and mainly focused on the coding sequence of the EGFR gene (exons 18, 19, 20, and 21). The NGS assay was conducted using the Illumina system (Illumina, USA), based on sequencing‐by‐synthesis technology. This strategy involves the incorporation of fluorescently labeled nucleotides into a growing DNA strand, one base at a time. Each incorporation event is detected by a high‐resolution imaging system, allowing for accurate determination of the DNA sequence. DNA libraries were accurately quantified using fluorescence‐based quantification methods. The percentage of reads on target and the percentage of regions below threshold were used to evaluate the quality of sequencing for each sample and the corresponding variant calls. Variant analysis was based on filtering, annotation, and data classification using data analysis software, allowing for identifying single nucleotide variants (SNVs) and insertions and deletions (indels).

### 2.4 AI Methodology

The proposed methodology aims to automatically detect EGFR‐mutated tissue within a WSI by utilizing a transfer learning approach with two CNNs. These two CNNs can autonomously identify optimal features from raw images, unlike traditional machine learning methods that rely on manually crafted features. A typical CNN consists of various elements, such as convolutional layers, activation layers, pooling layers, and fully connected layers. Training deep architectures with millions of parameters demands significant computational power, extensive memory resources, and time. To tackle these challenges, transfer learning is commonly employed. In transfer learning, a pre‐trained model is adapted or fine‐tuned for the specific task of interest. The last few layers of the network, responsible for task‐specific details, are adjusted to fit the new data.

**FIGURE 1:**
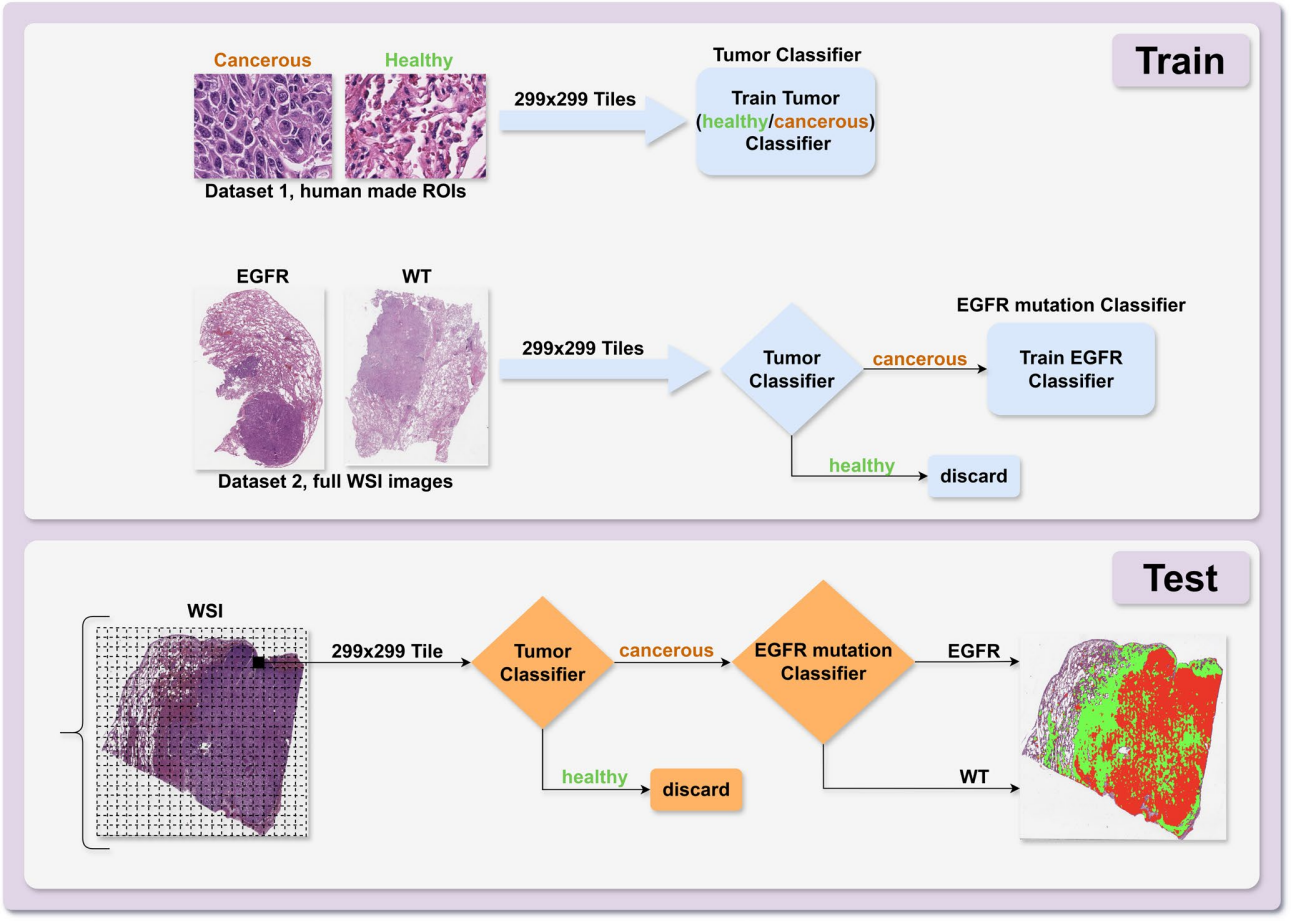
Upper panel: Workflow of the AI‐based methodology used to train the “Tumor Classifier” and the “EGFR Mutation Classifier”. Lower panel: Pipeline for applying the trained classifiers to a new WSI image. EGFR, Epidermal Growth Factor Receptor; ROI, region of interest; WSI, whole slide image; WT, wild‐type.

The specific methodological workflow of our work is depicted in Figure 1. Two different pretrained CNNs based on the InceptionResNet‐V2 architecture were utilized. Both CNNs were initialized with pretrained weights provided by MATLAB, obtained from training on the ImageNet‐1k dataset. The first CNN (Tumor Classifier) is designed to discriminate between cancerous and healthy tissue, while the second one (EGFR mutation Classifier) aims to detect EGFR mutation‐expressing tissue from WT tissue.

**FIGURE 2:**
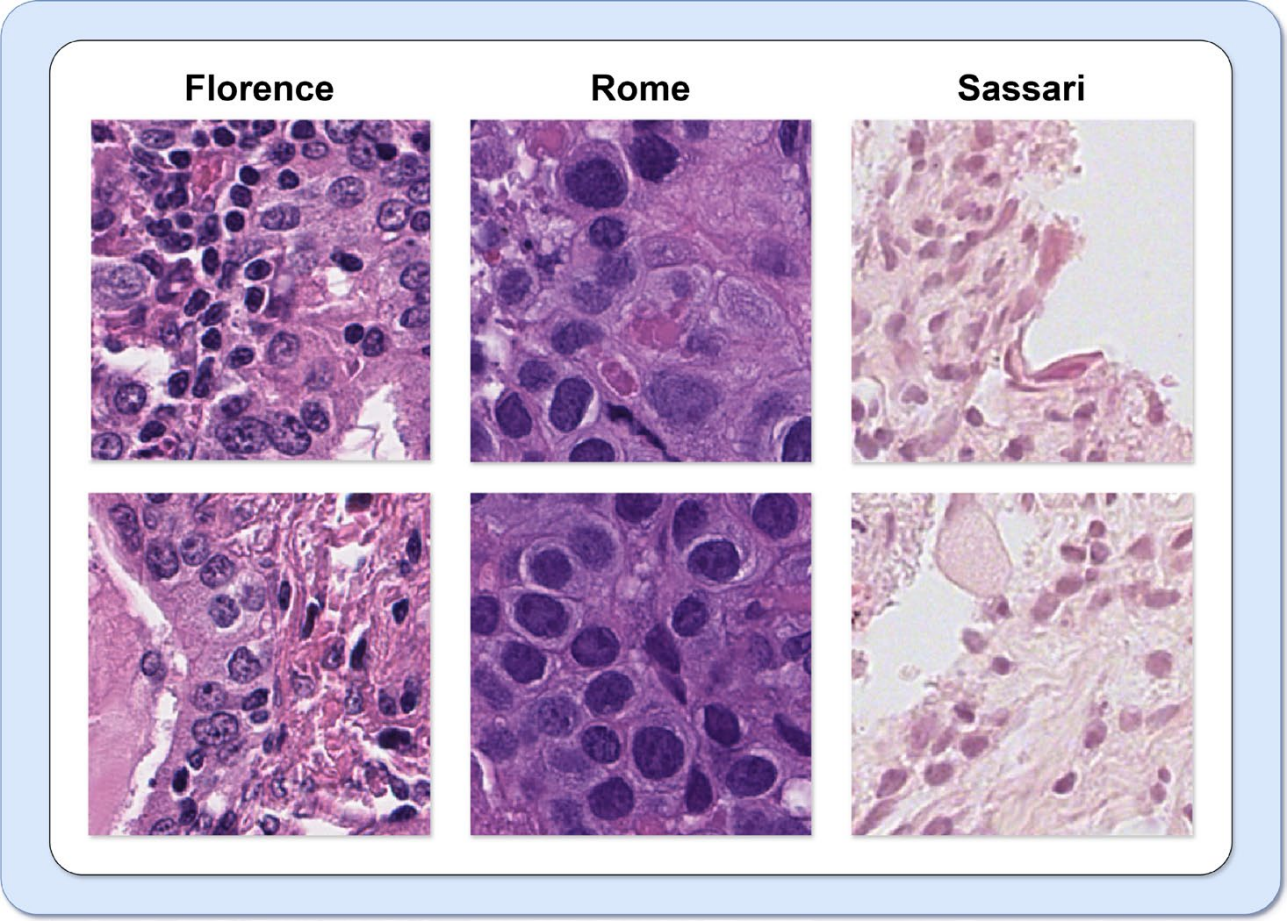
Heterogeneity of histological image tiles across the three centers (Florence, Rome, and Sassari).

The WSIs were acquired from 259 unique patients across three distinct clinical sites. These images were divided into a training subset (170 WSIs), a validation subset (29 WSIs) and a testing subset (60 WSIs). This partitioning ensured the establishment of independent datasets for training and testing phases, with each subset containing samples from different patients. Since the images from the three institutions exhibited a high degree of heterogeneity, particularly in staining, this variability was partly due to differences in sample preparation techniques across centers. For instance, the thickness of tissue sections varied from one center to another, which in turn affected the staining intensity and overall tile appearance as illustrated in Figure 2. We split the patient data into training, validation, and test sets independently of the institution of origin. This approach, based on random pooling and sampling from all sites, was chosen to ensure a balanced representation of inter‐site variability across all subsets and to improve the model's robustness against such heterogeneity.

For the training of Tumor Classifier, tiles from Regions of Interest (ROIs) at 40× magnification were manually selected by expert histopathologists. These ROIs served as the basis for extracting 299 × 299 pixel tiles for both the training and testing set. The tile size was selected to match the input of the InceptionResNet‐V2 architecture, thereby avoiding the need for additional resizing operations that could alter histological details. For the training of EGFR mutation Classifier, tiles were directly extracted from the WSIs, undergoing the same preprocessing applied to Tumor Classifier. The WSIs were partitioned into tiles, and each tile underwent individual classification by Tumor Classifier. Only the tiles that Tumor Classifier identified with a probability of at least 95% as cancerous tissues were included in the subsequent classification training set for EGFR mutation Classifier.

Given the intratumoral heterogeneity of EGFR mutations, it is not possible to determine whether each individual tile contains EGFR‐mutated tumor cells. As not every part of the tumor necessarily carries this specific mutation, this tile‐level labeling strategy inevitably introduces some degree of noise. To mitigate this, a set of 1000 example tiles was reviewed by experienced pathologists to ensure adequate representation of malignant regions. Moreover, the use of a large number of tiles per slide—combined through a majority voting mechanism for the final slide‐level prediction—was designed to reduce potential bias and better reflect the overall mutational status. While this introduces limitations in the classifier's performance at the tile level, the strategy was intended to prioritize robustness and clinical relevance of the slide‐level predictions, acknowledging the inherent biological and methodological complexity of the task.

Given that the images are sourced from various clinical centers, a preprocessing step is applied to enhance the standardization and generalization of the method. This involves stain normalization on the extracted images. These procedures work to mitigate differences in image coloration and enhance the distribution of contrast, contributing to a more consistent and robust analysis. To further enhance the training set, data augmentation was applied, utilizing the parameters outlined in Table 1.

**TABLE 1:** Data augmentation for classifiers.

Training	Geometric augmentation			Color augmentation			
	Pixel range	Scale range	Rotation	Hue	Saturation	Brightness	Synthetic blur
Tumor Classifier	[−10,10]	[0.5, 1.5]	[−90°, 90°]	—	—	—	—
EGFR mutation Classifier	[−10,10]	[0.5, 1.5]	[−90°, 90°]	[0.03, 0.04]	[−0.05, 0.05]	[−0.1, 0.1]	*σ* = 1 + rand

Both classifiers were trained using a learning rate of 0.001 and the SGDM optimizer (Stochastic Gradient Descent with Momentum), with the default momentum value of 0.9. This optimizer is well‐suited for fine‐tuning deep neural networks due to its convergence properties and stability. The loss function employed in both cases was the standard binary cross‐entropy loss, which is widely used in classification tasks as it effectively quantifies the dissimilarity between predicted probabilities and true binary labels.

In both the CNNs, we used the InceptionResNet‐V2 architecture as the backbone network. For the Tumor Classifier, we froze 471 layers and used a batch size of 128; for the EGFR Mutation Classifier, we froze only 1 layer and adopted a batch size of 64. This difference in the number of frozen layers reflects the disparity in the size of the training datasets. Specifically, the Tumor Classifier was trained on a relatively small number of tiles, which necessitated freezing most of the network to reduce the risk of overfitting. In contrast, the EGFR Mutation Classifier was trained on a much larger dataset, comprising tiles extracted from entire whole slide images (WSIs), which allowed for extensive fine‐tuning of the model and required freezing only the earliest layer of the network.

Training was performed on an NVIDIA RTX 3090 (24 GB VRAM) with 112 GB RAM and an AMD EPYC 7443P CPU under Windows (Table 2).

**TABLE 2:** Learning parameters for classifiers.

Training	Hyperparameters			Training time
	Frozen layers	Learning rate	Mini‐batch size	
Tumor Classifier	471	0.001	128	12 h
EGFR mutation Classifier	1	0.001	64	73 h

In the initial step of testing procedure, each tile undergoes classification by Tumor Classifier. If it is designated as cancerous, it further undergoes classification by EGFR mutation Classifier to evaluate the specific expression of the mutation. By the end of this process, each tile is assigned a class, categorizing it as healthy, EGFR cancerous, or WT cancerous.

After the completion of EGFR/WT classification over the tiles, the reassembly of each WSI enabled the evaluation of the AI‐mutation Ratio on a slide‐by‐slide basis, obtained by dividing the number of EGFR and WT tiles. The distributions of AI‐mutation Ratios in WSIs were statistically compared between the EGFR and WT groups of the testing dataset using a Wilcoxon test. Across every WSI, the AI‐mutation Ratio is derived through a voting process. This ratio represents the proportion of tissue identified as EGFR to tissue identified as WT within the slide. A classification threshold of 25% on the AI‐mutation Ratio was used to categorize each WSI as EGFR or WT. This reduced threshold, compared to standard majority voting, was selected to account for intratumoral heterogeneity and the limited presence of mutation features across tumor tiles.

The heatmaps reporting the spatial distribution of EGFR and WT tissue regions over each WSI were generated. To mitigate potential noise and spurious misclassified patches, a 3 × 3 median filter was applied. To evaluate the performance of both classifiers, multiple metrics—including accuracy, specificity, sensitivity, Cohen's kappa, and F1 score—were assessed following the methodology outlined in [38].

### 2.5 Model Explainability via High‐Dimensional Class Activation Maps

To investigate the decision‐making process of the EGFR mutation classifier, we selected a subset of input tiles on which the model had made correct predictions for both EGFR‐mutant and wild‐type (WT) classes. These tiles were ranked according to the classifier's predicted probability, and those with the highest confidence scores were retained for further analysis, ensuring that interpretability was assessed under conditions of maximal model certainty. High‐Dimensional Class Activation Maps (HD‐CAMs) were then computed for these selected tiles [39]. Specifically, HD‐CAMs were generated using a multilayer Grad‐CAM pipeline applied to the InceptionResNet‐V2 model trained for EGFR mutation status classification. Grad‐CAMs were extracted from the last ten ReLU activation layers, individually normalized, and upsampled to the original input resolution. These activation maps were subsequently combined using a weighted summation scheme, in which deeper layers were assigned higher weights via a linear distribution to emphasize increasingly abstract feature representations. The resulting HD‐CAMs provide multi‐scale, fine‐grained visual explanations of the model's predictions. A subset of these visualizations was reviewed by expert pathologists to qualitatively assess the correspondence between the highlighted regions and histopathologically relevant features, with the aim of evaluating the biological plausibility of the model's learned representations.

## 3 Results

### 3.1 Dataset Description

**FIGURE 3:**
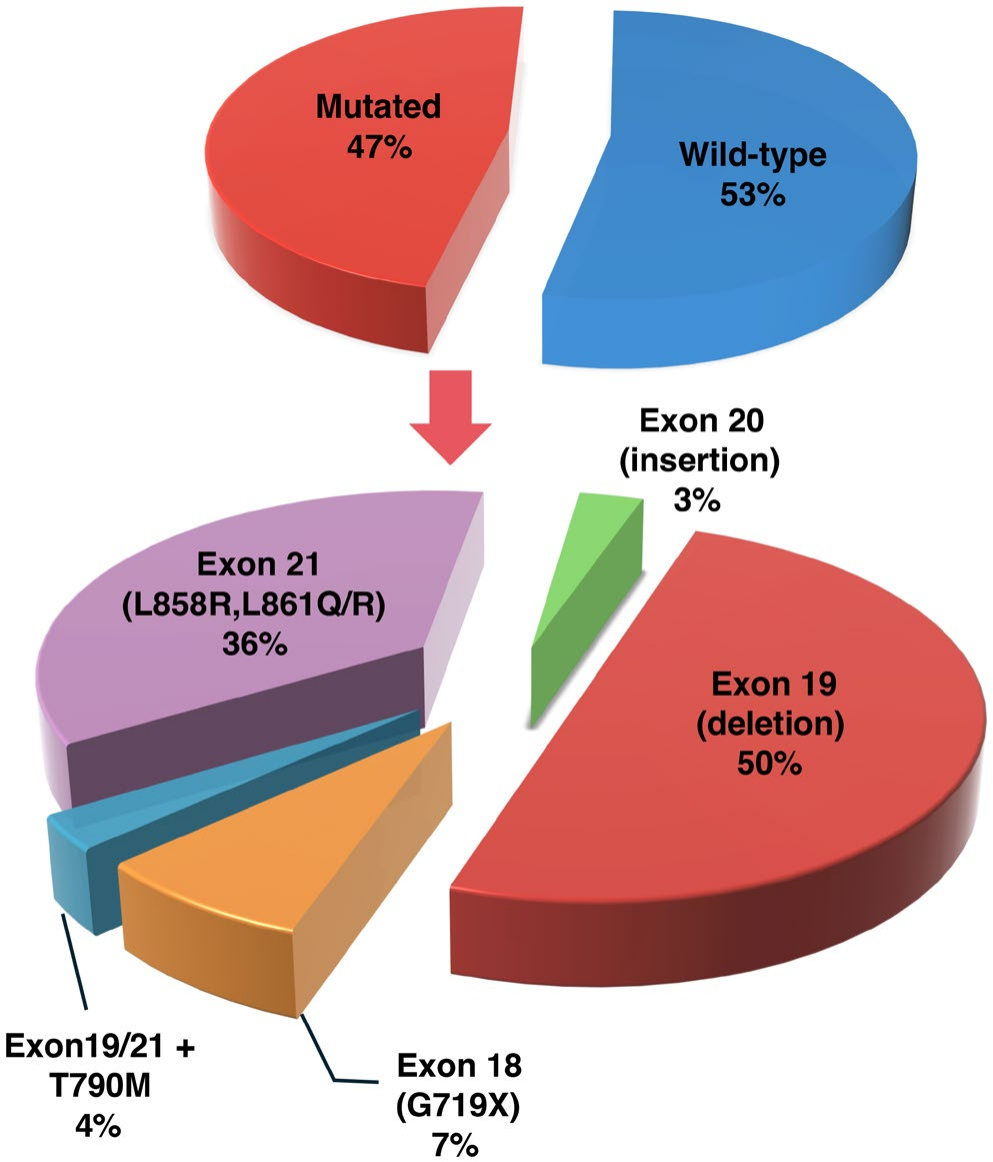
EGFR mutational status and frequencies of the different mutations.

Among the 259 FFPE ADC tissue samples collected in our study, 198 (76.45%) were biopsies and 61 (23.55%) surgical; EGFR mutations were present in 121 (47%) lung adenocarcinomas. Therefore, a comparable distribution of mutated and wild‐type (WT) EGFR cases (47% vs. 53%) was achieved in our series. Among EGFR mutants, deletions in exon 19 were the most representative alterations (60/121; 50%), followed by the mutations in exon 21 (44; 36%), at leucine in position 858 (L858R: 36) and at position 861 (L861Q: 7, L861R: 1). The remaining ones were mutations G719X in exon 18 (9; 7%) and an insertion in exon 20 (3; 3%). Five (4%) lung adenocarcinomas presented a more heterogeneous EGFR mutational status: three cases with concomitant tyrosine–kinase inhibitor (TKI)‐sensitive deletion in exon 19 and the TKI‐resistant T790M mutation in exon 20, and two cases with simultaneous occurrence of the TKI‐sensitive mutation L858R in exon 21 and the T790M variant. Overall, the vast majority—more than four fifths—of EGFR‐mutated cases included in our series presented an oncogenic driver alteration in exon 19 or exon 21. In Figure 3 is schematically represented the distribution of samples in our series according to the EGFR mutational status.

### 3.2 Cancerous vs. Healthy Tissues Classification

**FIGURE 4:**
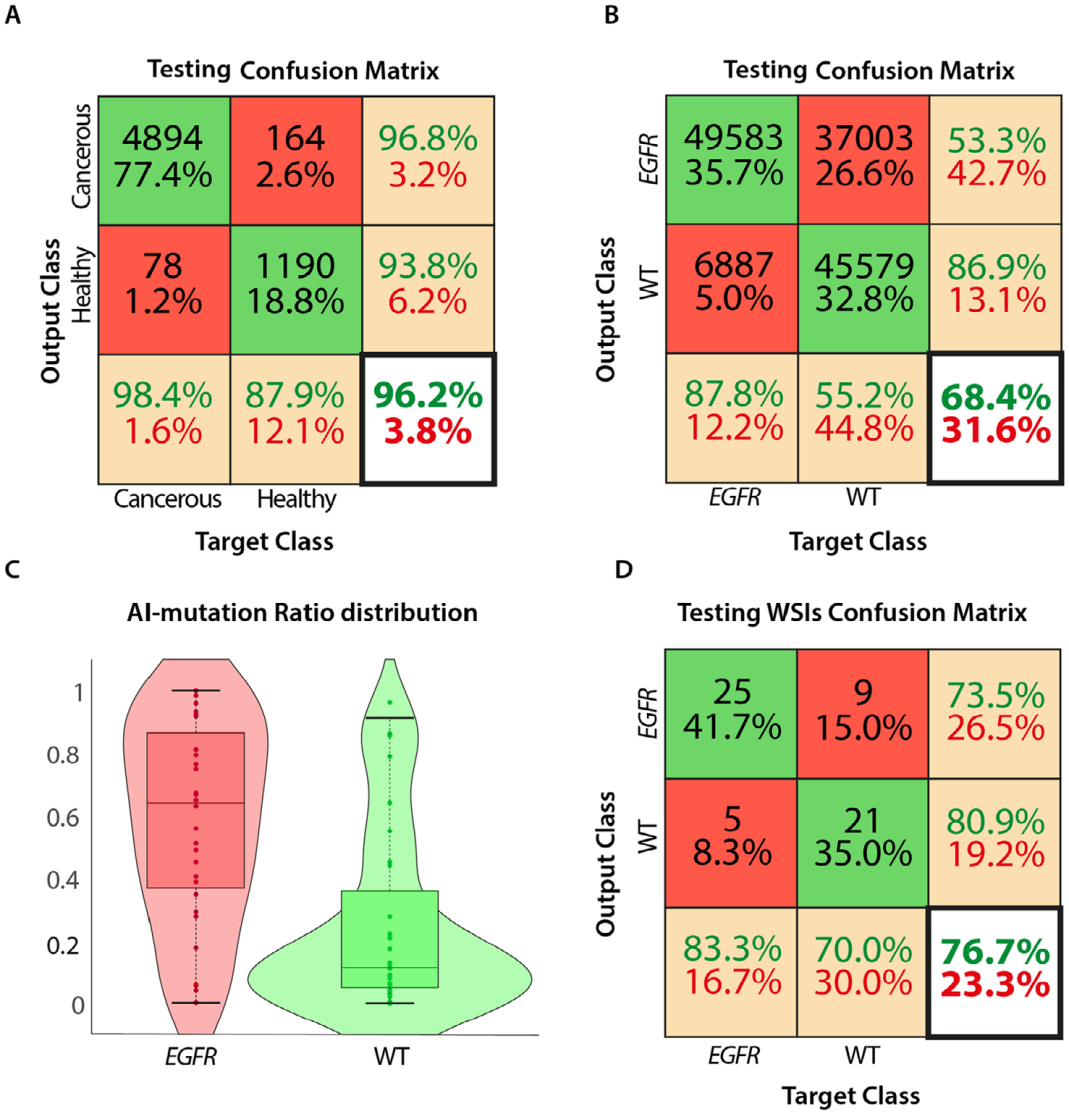
(A) Confusion matrix reporting the results of tiles classification on the testing set for Tumor Classifier. (B) Confusion matrix reporting the results of tiles classification on the testing set for EGFR mutation Classifier. (C) Boxplots and Violin plots reporting the AI‐mutation Ratio distribution for EGFR and WT WSIs. The two distributions are statistically different (Wilcoxon test, *p* = 0.0034). (D) Confusion matrix reporting the results of WSI classification on the testing set.

During the first step analysis of the 259 WSIs with the pretrained CNN model (Tumor Classifier), which is designed for the automatic analysis of histopathology images by discriminating between cancerous and healthy tissue, tiles of ROIs were extracted from both tissues, as shown in Table 3. The performance, considering votes with a confidence over 90%, outcomes of this analysis yielded an accuracy of 0.9617, a specificity of 0.8789, a sensitivity of 0.9843, an F1 score of 0.9759, a Cohen's Kappa of 0.7982 and an AUC of 0.989. Additionally, the confusion matrix for the test set classification, reported in Figure 4A, indicates that our network correctly classified 96.2% and misclassified 3.8% of the cases.

**TABLE 3:** ROI and extracted tiles numerosity used for Tumor Classifier.

Tissue	ROI	Extracted tiles (train/validation/test)
Healthy	4407	4188 (2265/655/1268)
Cancerous	5981	22,293 (14,266/2969/5058)

Abbreviation: ROI, region of interest.

### 3.3 EGFR vs. WT Cancerous Tissues Classification

The WSIs containing tiles that Tumor Classifier identified as cancerous were subsequently included in the second classification training set for EGFR mutation Classifier. Table 4 provides an overview of the number of WSIs and tiles obtained for the two classes, both before and after the exclusion of healthy tiles.

**TABLE 4:** WSI and correspondent extracted tiles numerosity.

Mutational status	WSI (train/validation/test)	Tiles obtained (train/validation/test)
EGFR	121 (77/14/30)	268,204 (122,947/24,750/120,507)
WT	138 (93/15/30)	203,654 (116,318/20,797/66,539)

Abbreviations: EGFR, Epidermal Growth Factor Receptor; WSI, whole slide image; WT, wild‐type.

On the tiles test set, considering votes with a confidence over the 90%, the following performance metrics score achieved an accuracy of 0.6844, specificity of 0.5519, sensitivity of 0.8780, F1 score of 0.6932, and Cohen's Kappa with a consistency of 0.4217. The Figure 4B demonstrates a confusion matrix of the test set EGFR mutation Classifier indicating a correct classification with 68.5%. The boxplots in Figure 4C depict the AI‐mutation Ratio across two WSI groups: patients with EGFR‐mutated cancerous tumor and those with WT cancerous tumor. The result of a statistical analysis using the Wilcoxon test reveals a significant difference between the distribution of these two groups (*p* = 0.0034).

Based on the use of AI‐mutation Ratio from the voting process, the confusion matrix in Figure 4D demonstrates a correct classification rate of 76.7% and a misclassification rate of 23.3%. By setting the classification threshold at 25% for the AI‐mutation Ratio, the model obtains the following performance metrics: accuracy of 0.7667, specificity of 0.8077, sensitivity of 0.7353, F1 score of 0.7812, and Cohen's Kappa of 0.5583. Moreover, the corresponding WSI‐level AUC reaches 0.77.

**FIGURE 5:**
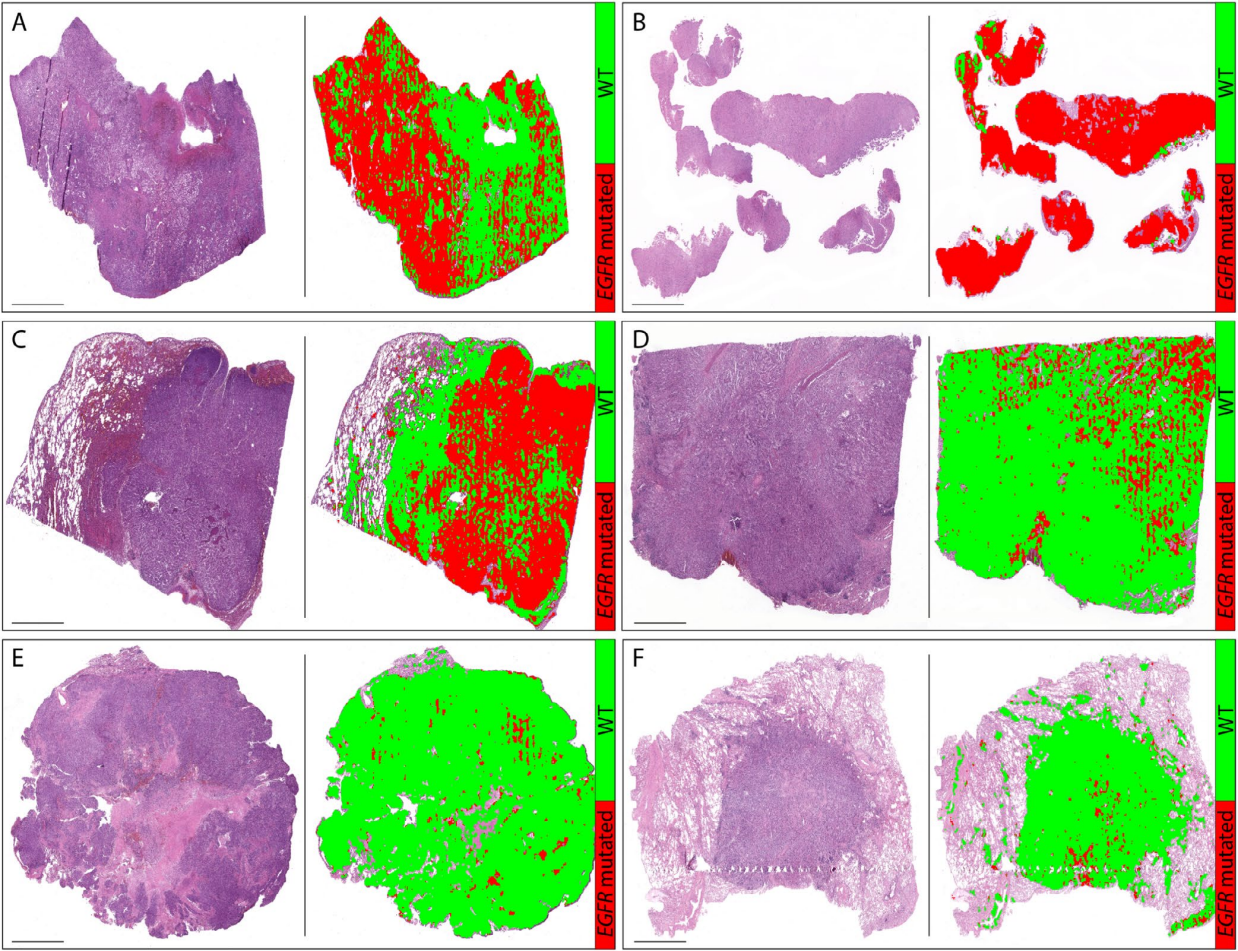
Examples of heat maps for predicting EGFR and WT mutational distribution: (A) EGFR WSI, AI‐mutation Ratio = 63%. (B) EGFR WSI, AI‐mutation Ratio = 98%. (C) EGFR WSI, AI‐mutation Ratio = 76%. (D) WT WSI, AI‐mutation Ratio = 13%. (E) WT WSI, AI‐mutation Ratio = 4%. (F) WT WSI, AI‐mutation Ratio = 3%.

The Figure 5 illustrates examples of heatmaps produced by the model to visualize the spatial distribution of healthy tissue, EGFR‐mutated, and wild‐type tumor areas across different H&E‐stained WSIs. In EGFR‐positive cases (A, B, and C), most of the tumor regions are highlighted in red and these often correspond to areas showing dense tumor growth, nuclear atypia, or solid patterns, common features that are observed in EGFR‐mutant adenocarcinomas. In contrast, wild‐type cases (D, E, and F) are mostly marked in green, with a few red areas that might reflect regions of morphological ambiguity or subtle histological variation. These visualizations highlight how the model captures slight histological differences associated with mutational status and suggest that it may be detecting some meaningful histological cues, even without explicit region‐level labeling.

**FIGURE 6:**
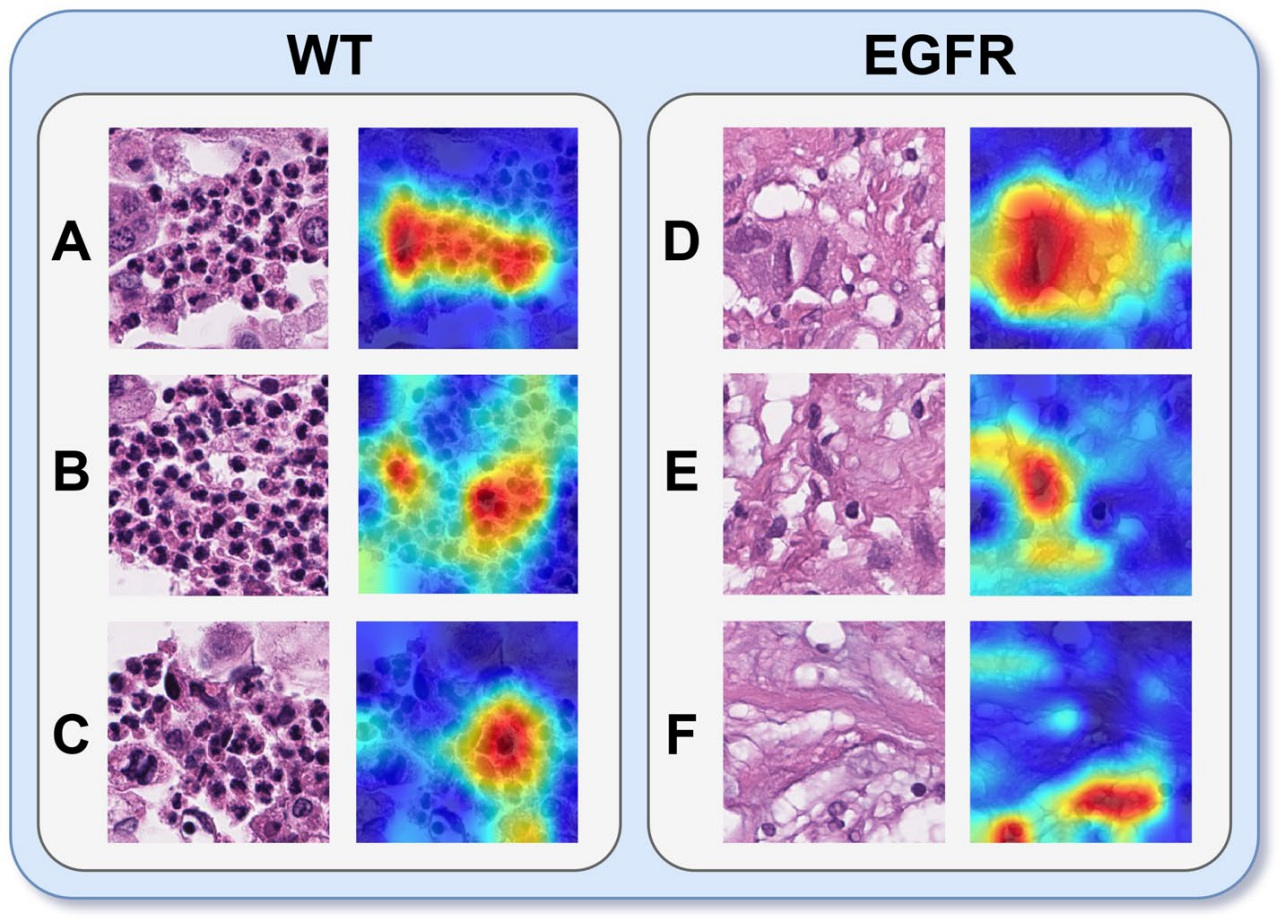
HD‐CAM visualizations for WT and EGFR‐mutant samples. H&E‐stained histology images (left) and corresponding HD‐CAMs (right) are shown for wild‐type (WT, A–C) and EGFR‐mutant (D–F) tiles.

Figure 6 displays representative tiles from EGFR‐mutated and WT cases, each shown with its corresponding HD‐CAMs. The resulting HD‐CAMs displayed well‐localized and spatially coherent activation patterns, indicating that the network focused on structured and semantically relevant features during the classification process. In the visualizations, red regions correspond to areas with strong positive contributions to the predicted class, highlighting features that the model considered most discriminative. Conversely, blue regions indicate areas with little or no influence on the classification decision, or in some cases, negative contributions.

From a clinical perspective, the WT group (Figure 6A‐C) shows tumor and apoptotic cells within a necrotic background in panels (A) and (B), while there is a necrotic area with a visible mitosis in panel (C). In contrast, the EGFR‐mutated samples (Figure 6D‐F) reveal nuclear pleomorphism in panel (D), along with atypical and irregular nuclear morphology in panels (E) and (F). These patterns suggest that the model's attention focuses on biologically meaningful regions, supporting the interpretability of its predictions from both technical and clinical standpoints.

## 4 Discussion

In recent years, it has been demonstrated that in medicine, the use of AI presents several advantages, including standardization and reproducibility, improvements in diagnostic accuracy and efficiency with lower overall time‐consuming costs [40].

Currently, the advancement of WSI technology has been accompanied by the rapid progress in image processing methods, emerging as a potent tool for the analysis of digital pathology images, especially with the integration of AI and deep learning, which has sparked significant transformation and debate within the field. In this study, we developed a model utilizing a transfer learning approach with two CNNs based on the InceptionResNet‐V2 and having the ability to identify optimal features from raw images to differentiate between tumoral and healthy tissues and to predict EGFR mutations from tumoral tissues based on FFPE‐259 WSIs that were collected from 259 patients. Our data showed that our AI CNN model that automatically recognizes lung ADC from nonneoplastic tissue could be a useful diagnostic tool for assisting pathologists with diagnosis. It demonstrated a promising performance in detecting cancerous areas in histopathological slides of both biopsy and surgical specimens by recognizing portions of pathological and healthy regions on independent testing datasets. The results achieved a high accuracy, specificity, and sensitivity (96.17%, 87.89%, and 98.43% respectively), in addition to a high performance with a correct classification of 96.2% and an AUC of 0.989. This first result is in line with the most recent data provided by the literature.

Several research groups have explored algorithms to differentiate between tumoral and healthy tissues using various CNN models. For instance, Matko et al. used VGG16 and ResNet50, reporting accuracies between 72.05% and 75.41%, with an AUC of 0.86. Gupta et al. applied ResNet50 with CLAM (Clustering‐constrained Attention Multiple Instance Learning) and achieved an AUC of 0.964. Kriegsmann et al. tested VGG16, InceptionV3, and InceptionResNetV2, where the accuracy ranged from 83% to 95%. This range of performance highlights both the diversity of deep learning techniques in pathology and their evolving capability to support tissue classification tasks. Our model fits within this landscape, showing a strong performance in distinguishing healthy from cancerous tissue, also reinforcing the potential of CNN‐based solutions in digital pathology [41, 42, 43]. At the same time, some authors have hypothesized that the low failure rate in recognizing healthy versus neoplastic tissue (false negative or false positive cases) is generally due to three main factors: first, confounding morphological features, such as intra‐alveolar macrophages or reactive bronchial cells recognized as neoplastic cells, or conversely, cellular elements with minimal atypia recognized as nonneoplastic; second, the use of different sample preparation and staining methods; third, the use of different scanners. To overcome these limitations, it is necessary to train these technologies with a large number of cases representing these variations [30].

The second aim of our study was to observe how our architectural model was able to predict the mutational status of EGFR in NSCLCs. The initial results presented on the tiles exhibit a suboptimal performance with an accuracy of 68.44%, specificity of 55.19% and sensitivity of 87.80%, along with a misclassification rate of 31.6% (Figure 4B). By aggregating the tiles according to their WSI and computing the AI‐mutation Ratio distribution for EGFR and WT, we obtained an improved performance, with a statistically significant difference between the EGFR‐mutated and EGFR‐wildtype observed groups (Wilcoxon test *p* = 0.0034). Further improvement was achieved through the implementation of a voting procedure applied on the aggregated results, yielding several key performance metrics, including accuracy of 76.67%, specificity of 80.77%, sensitivity of 73.53%, F1 score of 78.12%, and Cohen's Kappa of 0.5583, along with a lower misclassification rate of 23.3% (Figure 4D). The corresponding WSI‐level AUC is 0.77. These findings support the reliability of the model for the noninvasive EGFR mutation prediction in lung adenocarcinoma, showing a notable enhancement in predictive performance after aggregation. Various methods have been proposed to predict EGFR mutation status, and all have produced promising results. Zhao et al. used a ResNet50 model and presented an accuracy of 75%, with an AUC of 0.82, specificity of 74%, and sensitivity of 76% [44]. Wang et al. developed the HEAL framework, which integrates 16 deep learning architectures, and reached an AUC of 0.82 as well [45]. Pao et al. also tested ResNet50, reaching an AUC of 0.87 [46], while Gupta et al. reported an AUC ranging from 0.78 to 0.864 [42]. In this context, these findings are comparable to our results and further support that EGFR mutation can be detected from routine H&E slides using deep learning. In some cases, a distinctive approach has been employed to improve predictive accuracy. In a study conducted by Jiang et al. clinical data were integrated with both semantic and spatial features of images. By combining these different information sources, the researchers suggested that their method significantly improved predictive power, providing a better comprehensive understanding of the factors that influence mutational status [47]. Interestingly, there have been described more complex models able to predict not only the EGFR status, but the most mutated genes in lung ADC (BRAF, EGFR, KRAS, STK11, TP53, FAT1, SETBP1) demonstrating promising outcomes, however with more variable accuracy ranges (AUC of 0.45–0.856) [29, 48].

An important finding of our study is that the diagnostic sensitivity exceeds that reported in previous research. This improvement underscores the effectiveness of our approach and suggests a greater ability to accurately identify positive cases. Previous studies proposed deep learning models trained on CT images to predict EGFR mutational status in LC with equally promising results [49, 50, 51]. As already observed in pathology, some authors have shown that the approach with fusion models integrating clinical, radiological, radiomic, and deep learning features presented superior results compared to models using only single data. By combining different types of data, the fusion model was able to capture a more complete picture of the disease, improving diagnostic and predictive accuracy [52].

In this study, we used the Grad‐CAM visualization technique to visually explain the CNN model's prediction process by showing alignment between morphological patterns and pathological features. This helped us better discriminate class‐specific regions that contributed to the prediction of EGFR mutation, adding an interpretability layer that is increasingly recognized as essential in clinical AI applications. Previous studies demonstrated that integrating several explainability techniques, such as Grad‐CAM, Grad‐CAM++, and Score‐CAM into deep learning workflows, revealed the gap between model complexity and practical utility in diagnostic settings, thereby improving trust and understanding of CNN‐based decisions. These attention maps enhanced model transparency and supported clinicians in relating AI outputs to meaningful histological signals [53, 54]. Such applications were also explored in predicting EGFR mutation status through CT imaging [55]. Taken together, after completing the recognition and detection process in the WSIs, the generated heat maps which represent spatial distributions prove to be a valuable tool capable of aiding various aspects of the pathologist's evaluation. They can effectively locate the specific regions correlated with tissue classification, including EGFR‐mutated cancerous tissue and WT cancerous tissue, making them well‐suited for automating the analysis process. Overall, this model shows its potential as a highly useful tool for assisting pathologists in classifying lung tissue WSIs, providing crucial prognostic information for tumor staging and aiding in the application of appropriate targeted therapy. The obtained classification performance does not exceed the best results reported in literature but confirms the potential to develop automatic recognition methods that maintain effectiveness across different clinical centers, proving the high generalization capability of the proposed model in a real clinical setting. However, our model demonstrated an increased sensitivity, which might be advantageous for clinical settings that emphasize mutation detection over avoiding false positives. Our model's sensitivity suggests that it could serve as an important tool in the early identification of these mutations, thus facilitating timely therapeutic interventions.

While the CNN models have shown encouraging outcomes, an important limitation arises from the small sample size used for training and testing, which may limit the generalizability. Additionally, the weakly supervised labeling strategy introduces noise, which affected the EGFR mutation classification performance, reflecting methodological challenges due to intratumoral heterogeneity. To reduce the risk of false negatives, a conservative voting threshold of 25% was adopted, based on the distribution of tile predictions. The current model was designed for binary classification (EGFR‐ mutated vs. WT), which does not account for the diversity of mutation subtypes.

In future work, it will be important to conduct studies with a larger sample size dataset and to develop an adopted model capable of incorporating more detailed mutation subtypes that can guide targeted therapies. Using finer annotation strategies or frameworks such as Multiple Instance Learning (MIL) could also help capture global context and improve tile‐level precision and label aggregation, which will allow us to focus on localized patterns and offer a promising direction for future studies. Further evaluation is needed to understand how such models could be integrated into real‐world workflows, particularly for prioritization of molecular testing in resource‐limited settings. These steps, along with prospective validation, will be essential for refining the model's clinical utility. Yet, it would be interesting in future work to conduct studies with a larger sample size and to develop an AI approach to detect other molecular alterations such as ALK, TP53, or KRAS. Importantly, predicting EGFR gene mutational status would further advance digital pathology through AI applications. Integration of AI models like ours into routine clinical workflows could significantly reduce the time and cost of molecular diagnostics. While current techniques like NGS are accurate, to date they are time‐consuming and expensive. An AI‐based system could serve as a rapid prescreening tool to identify candidates for more detailed molecular testing. Furthermore, as previously reported, most of the available AI tools have been tested and validated on retrospective data, which do not represent real‐world scenarios. It will be important in the future for the use of these tools in clinical practice to be able to validate algorithms in randomized clinical trials and prospective studies. Therefore, the proposed approach offers significant opportunity for improving precision in oncology prediction; thereby it would be beneficial for targeted therapy and personalized treatment. In the future, these approaches could be improved and utilized as prescreening tools to select samples for sequencing, assess potential clonality or heterogeneity, and gain deeper insights into how genetic alterations affect function and morphology.

## 5 Conclusion

In summary, our study was able to demonstrate that the use of these two different pretrained CNN models, based on the InceptionResNet‐V2 architecture, has achieved promising results. On one hand, they are generally satisfying for tumoral tissue detection, and on the other hand, they illustrated encouraging outcomes for predicting EGFR and WT mutation on WSIs. By incorporating clinical features, pathological CNN algorithms can be valuable for diagnosing WSIs and biomarker analysis. Nonetheless, they have the potential to enhance workflow efficiency for pathologists and increase the performance of medical precision, particularly in LC.

## Author Contributions

All authors participated in the writing, reviewing, and editing of the manuscript and have approved the final version of the paper.

## Ethics Statement

The present study was conducted on FFPE (formalin‐fixed paraffin‐embedded) sections of anonymized human samples and was carried out in accordance with the principles of the Declaration of Helsinki, under the approval of the Local Ethics Committee 20753_bio and the Committee for the Ethics of the Research and Bioethics of the National Research Council/CNR n.12629. All patients were fully informed of the aims and methodology details regarding the use of their tissue samples and provided written informed consent.

## Conflicts of Interest

The authors declare no conflicts of interest.

## Data Availability

All data/information used and analyzed during this study are available upon reasonable request to the corresponding author.
